# The Silent Saboteur: Teaching the Clinical Implications of Occult Hypoxemia & Social Determinants of Health via a Pulmonary Embolism Case

**DOI:** 10.21980/J8FD14

**Published:** 2025-04-30

**Authors:** Eugene Marrone, John Cafaro, Jared Klein

**Affiliations:** * Cooper Medical School of Rowan University, Cooper University Health Care, Department of Emergency Medicine, Camden, New Jersey

## Abstract

**Audience:**

Medical students on required fourth-year emergency medicine clerkship

**Introduction:**

Social determinants of health are the nonmedical factors that influence health outcomes.^1^ As part of the AMA Accelerating Change in Medical Education Consortium's third pillar of medical education, health systems science, social determinants of health are recognized as critical components to medical student education.^2^ The push for institutions to address health inequities has led medical schools to emphasize social determinants of health.^3^ Medical students have stepped up to advocate for change and are demanding concrete action, including the development of antiracist curriculum and learning environments.^4^ The current and next generations of physicians need to be prepared to be responsive to the public health and societal needs of everyone.^5^ Emergency departments are a window into a community and its challenges, reflecting the most critical social determinants of health (SDH) of the population they serve; as such, they are the ideal setting in which to learn about SDH.^6^ Core emergency medicine (EM) clerkships typically focus on disease management for the acutely ill and injured, with limited emphasis on the holistic care that addresses a patient's SDH—a missed educational opportunity.^7^ We present an oral (or white) board case that highlights the basic approach to pulmonary embolism while emphasizing consideration of both social determinants of health and racial considerations.

**Educational Objectives:**

By the end of this oral board case, learners will be able to: 1) obtain appropriate history of present illness (HPI) and physical exam elements for the undifferentiated chest pain patient, 2) identify elements of history and physical exam that are compatible with pulmonary embolism, 3) formulate a differential diagnosis for chest pain and perform the appropriate work-up to narrow this differential diagnosis, 4) appropriately manage pulmonary embolism, 5) review and discuss the diversity, equity and inclusion (DEI) elements of the case, and 6) review and discuss the importance of social determinants of health (SDH) in disposition decisions and patient outcomes.

**Educational Methods:**

This case is meant to be used as an oral board or white board case for medical students and interns.

**Research Methods:**

Educational content was assessed via three questions related to occult hypoxemia and Glomerular Filtration Rate (GFR) reporting by race at the end of clerkship exam. The results of learners who were present for the case were compared to those who were not present. Results were stratified to compare whether the student was applying for an Emergency Medicine residency or another specialty.

**Results:**

A total of 72 students completed the end of clerkship exam, with three questions related to diversity, equity, and inclusion. Data was sorted both by questions and whether the student planned to apply for an Emergency Medicine residency. The total percent correct was 54.63%. The total percent correct for students present for the oral boards case was 54.69% while that of students who were not present for the case was 54.17% (p=0.96). When looking at students applying for emergency medicine, the total percent correct was 61.90% compared to 47.75% correct for students who were not applying for an Emergency Medicine residency (p=0.037).

**Discussion:**

This case demonstrates an original way to teach core emergency medicine content and meet AAMC Diversity, Equity, Inclusion competencies. The case not only provides a realistic example of downstream effects of racial disparities and not addressing a patient's social determinants of health, but effectively illustrates how to integrate knowledge of inequity into patient care.

**Topics:**

Undifferentiated chest pain, pulmonary embolism, PERC Score, Well’s Score, occult hypoxemia, racial bias in reporting glomerular filtration rate (GFR), social determinants of health, diversity, equity, inclusion (DEI).

## USER GUIDE

**Table:** 

List of Resources:	
Abstract	1
User Guide	3
For Examiner Only	8
Oral Boards Assessment	13
Stimulus	16
Debriefing and Evaluation Pearls	28

**Learner Audience:**

Medical students on required fourth-year emergency medicine clerkship

**Time Required for Implementation:**

Case: 60 minutes

Debriefing: 30 minutes

Learners per instructor: 1–5

**Topics:**

Undifferentiated chest pain, pulmonary embolism, PERC Score, Well’s Score, occult hypoxemia, racial bias in reporting glomerular filtration rate (GFR), social determinants of health, diversity, equity, inclusion (DEI).

**Objectives:**

By the end of this oral board case, learners will be able to:

1. Obtain appropriate HPI and physical exam elements for the undifferentiated chest pain patient.
2. Identify elements of history and physical exam that are compatible with pulmonary embolism.
3. Formulate a differential diagnosis for chest pain and perform the appropriate work-up to narrow this differential diagnosis.
4. Appropriately manage pulmonary embolism.
5. Review and discuss the diversity, equity and inclusion (DEI) elements of the case.
6. Review and discuss the importance of social determinants of health (SDH) in disposition decisions and patient outcomes.

### Linked objectives, methods and results

The primary objective of this case is to contextualize a common emergency department clinical encounter through a social determinant of health lens. Specifically, learner(s) are exposed to the clinical implications of occult hypoxemia and learn how to integrate this knowledge into their medical decision-making. In addition, this case highlights the importance of weighing a patient’s social determinants of health and accessibility to healthcare resources upon disposition by including a repeat presentation to the Emergency Department with a massive pulmonary embolism. The learner will also develop a basic approach to the undifferentiated chest pain patient and identify history and physical exam findings compatible with pulmonary embolism. The learner should feel comfortable managing pulmonary embolism and applying clinical decision rules with regards to work up, treatment, and disposition.

Learners will progress through the case by generating an emergency medicine-based differential diagnosis for chest pain, gather a differential diagnosis driven history, risk stratify the patient via clinical decision rules, and ultimately formulate a management plan. However, this case also demonstrates to the learners how to clinically integrate knowledge of racism in medicine, racial disparities, healthcare inequities, and social determinants of health into their overall medical decision-making and care for their patients. Specifically, the case easily transitions to discussion on the clinical implications of occult hypoxemia and glomerular filtration rate (GFR) reporting and various barriers patients must overcome to carry out their care plan.

The conceptual framework used to develop the content was the importance of the bio-social model of health. This model recognizes that health and illness are influenced not only by biological factors but also by social determinants. Exploring social determinants of health in the setting of a realistic patient encounter helps learners understand how factors such as access to healthcare, socioeconomic status, and both community and emergency department resources impact a patient's treatment options and outcomes. By conceptualizing this in an oral boards format, the learners’ understanding of medicine goes beyond the traditional biomedical approach, fostering a more holistic perspective on patient care.

The selection of the oral boards format for teaching this case was driven by its interactive nature, which promotes active learner participation and discussion. Flipped classroom settings allow for learner application of knowledge, critical thinking, problem-solving, active learning, and effective communication. In addition, this format allows for a more dynamic learning environment where students can actively engage, share their perspectives and collectively construct knowledge through meaningful discussion.

### Recommended pre-reading for instructor

#### Racial Bias in Pulse Oximetry

- The pulse oximeter uses differences in infrared (oxyhemoglobin) and red (deoxyhemoglobin) light absorption to indirectly measure blood oxyhemoglobin saturation.
- Poor circulation, **skin pigmentation**, skin thickness, skin temperature, fingernail polish can all affect the device’s accuracy.
  ○ Pigmentation likely changes the relationship between amount of light transmitted and the oxygen Level. 8
- NEJM study detected occult hypoxemia (< 88%) despite oxygen saturations of 92 to 96% on pulse oximetry device in 11.7% of black patients compared to 3.6% of white patients.^9^
- **Clinical Significance:** Occult Hypoxemia
  ○ We hospitalize patients for new oxygen requirements. A black patient with a pulse oximeter reading of 92% may be discharged, but that reading may be falsely elevated due to skin pigmentation. Thus, we are discharging a patient who is actually hypoxemic. This is dangerous.
  ○ **COPD:** oxygen cut offs are used to decide who requires home oxygen (SpO2 < 88% is one of the indications).

#### GFR Reporting in African Americans & Non-African Americans

- Race was initially included in the GFR calculation because clinical trials demonstrated African Americans, on average, have higher creatinine Levels. This was thought to be due to larger muscle mass and differences in diet. This, of course, was incorrect and lead to clinically important downstream effects.
- National Kidney Foundation (NKF) and American Society of Nephrology (ASN) formed a joint task force to develop a new race-free estimated GFR calculation.
- **Clinical Significance:** reduce access to kidney transplantation; delayed dialysis
  ○ **Non-black patient:** Cr 2.8, GFR 18 (lower GFR, more likely for transplant)
  ○ **Black Patient:** Cr. 2.8, GFR 21 (higher GFR, transplant may not be indicated)

#### Social Determinants of Health

- Barriers to outpatient care is a reasonable reason for hospitalization of a patient.
- **Cost:** On prescription assistance coupon sites, the Rivaroxaban starter pack is ~$750.
- **Access:** Distance from pharmacy and transportation is often an issue.
  ○ **Health Insurance:** a major factor in patient’s access to care.
- **Health Literacy**
- **Options in ED:** Social Worker and Prescription coupons (ED Pharmacists).

#### *Presenting this Patient:* “65-year-old African-American male…”

- Antiquated form of patient presentation from when hospitals were segregated.
- Racial categories are not equivalent to genetic ancestry. Race is a complex social and political construct that may engender bias.

### Results and tips for successful implementation

Medical students may be unfamiliar with oral boards format. The instructor should pre-brief the students on the formatting and flow of a typical oral boards case before the case is begun. There is an optional video example on American Board of Emergency Medicine (ABEM) that can be reviewed before the case is begun which is referenced below.^10^ The objectives of understanding clinical implications of occult hypoxemia and social determinants of health were tested at the end of the clerkship examination, three weeks after this case was conducted. A total of 72 fourth year medical students participated in our end of clerkship exam. Three questions on the examination were tested relating to DEI and SHD. The three questions asked are as below:

1. *A 60-year-old African American male presents to the emergency department with dyspnea and pleuritic chest pain. His blood pressure is 140/90 mm Hg, heart rate is 110 bpm, respiratory rate is 22 breaths per minute and pulse oximetry is 98%. His GFR is 60. He is diagnosed with a small subsegmental pulmonary embolism on CT angiography of his chest and discharged on a Factor Xa Inhibitor. His barriers to care include lack of health insurance and access to transportation. He stops taking his anticoagulation because of the high cost of the medication. He returns to the emergency department 2 weeks later in respiratory distress. His blood pressure is 70/35 mm Hg, heart rate is 135 bpm, respiratory rate is 28 breaths per minute and pulse oximetry is 84%. Which of the following is the best step in management on the first emergency department visit to decrease the chance of the patient’s decompensation?*
  - Discharge the patient on warfarin because it is a cheaper alternative
  - Discharge the patient on a Factor Xa Inhibitor with a prescription coupon card and ensuring he was able to fill the medication, with instructions to return to the ED if he is unable to adhere to the treatment plan
  - Hospitalize the patient due to concerns for occult hypoxemia
  - Hospitalize the patient and defer CT angiography for Lung Ventilation Perfusion Scan (V/Q scan) due to GFR being artificially increased due to race
2. *A 65-year-old African American female with a history of hypertension, asthma and chronic kidney disease (CKD,GFR = 25) presents to the emergency department with six days of fever, cough, body aches, nasal congestion and loss of taste and smell. She states she is short of breath and feels like she needs oxygen. Her pulse oximetry is 91%. She is wheezing bilaterally but that improves after three treatments of albuterol-ipratropium. Repeat vital signs show the same pulse oximetry of 91%. Chest X-Ray shows bilateral interstitial infiltrates. She is diagnosed with COVID-19. What is the most appropriate management?*
  - Discharge the patient with a handheld pulse oximeter, Albuterol inhaler, and a prescription for prednisone
  - Discharge the patient with a handheld pulse oximeter, Albuterol inhaler and a prescription for prednisone and doxycycline
  - Hospitalize the patient and start steroids
  - Discharge the patient with no medications and primary care physician (PCP) follow up
3. *Which of the following statements is true regarding eGFR?*
  - The use of race in eGFR calculations is supported by robust genetic evidence linking race to kidney function
  - It is medically appropriate to report different eGFRs
  - Reporting eGFR by race has no downstream clinical significance
  - Reporting eGFR by race can result in reduced access and/or delays in kidney transplantation and initiation of hemodialysis

The outcome measured was the percentage correct of the three DEI/SDH related questions on the examination. We stratified our results by comparing both students applying for emergency medicine residencies vs non-emergency medicine residencies. Additionally, we compared students who were present for the oral board case vs students who were not present. The total percent correct of the three DEI questions for all students who completed the exam, regardless of whether they were present for the case and regardless of their chosen subspecialty was 54.63%. The total percent correct for students present for the oral board case was 54.69% while students who were not present for the case was 54.17% (p=0.96). When looking at students applying for emergency medicine, the total percent correct was 61.90% in comparison to 47.75% correct for students who were not applying for an emergency medicine residency (p=0.037). Statistical significance was explored using a Chi-square analysis. While comparing students who were present for the case versus those who were not showed no statistical significance, those applying for an emergency medicine residency vs those who were not showed statistical significance. We hypothesize this may be due to the increased work ethic and rigor of the students applying for Emergency Medicine because their outcome of the clerkship will affect their standardized letter of evaluation which has a significant impact on residency applications. While our data is limited to a small sample size, initiating a dialogue on this important area holds promise for the growth and advancement of future medical professionals.

The subject of DEI within the field of medicine is extensive and cannot be adequately addressed by a single oral boards case. The American Academy of Medical Colleges (AAMC) released new cross-continuum competencies in 2022 to help educators design and deliver curriculum regarding DEI while helping learners with their individual professional development. 5 Many medical schools have incorporated medical education program objectives on racism in medicine and social determinants of health to their current curricula.^5^ This oral boards case addresses these objectives with a case clinically relevant to emergency medicine.

Many faculty members have expressed a desire and need to develop their skills so they can be better equipped to facilitate discussions on race and racism. This underscores a gap between learners and faculty on social justice issues. Current students and residents compared with faculty demonstrate a greater knowledge of social justice topics such as the history of racism in medicine, structural and institutional racism, structural competency, socioecological determinants of health, and health inequities.^2^ This is likely because these topics are now being taught more in medical schools and residency. However, emergency medicine faculty consistently advocate for healthcare equity of their patients through various modalities such as utilizing community and emergency department resources and are perfectly equipped to facilitate this case.

While this case can be utilized with individual learners, small group settings offer enhanced value through dynamic student discussion, interaction, and engagement. By implementing the case in groups of three to four students, we fostered thoughtful dialogue on both the medical management aspects and the social and public health disparities addressed in the case. This format also creates a unique opportunity for students, residents, and instructors to share personal experiences and case stories related to the impact of social determinants of health in the Emergency Department.

### Pearls

Common symptoms of pulmonary embolism (PE) include sudden chest pain, shortness of breath, palpitations, and hemoptysis. The Well's score is a clinical decision rule used to assess the probability of a patient having a PE^11^. It aids in identifying patients who require additional investigations, such as imaging with computed tomography pulmonary angiography (CTPA) or V/Q scanning, to confirm or exclude the presence of a PE. A Well’s score is comprised of clinical signs and symptoms of DVT, PE is the most likely diagnosis, tachycardia, recent surgery or immobilization, previous PE or DVT, hemoptysis, and malignancy. Altogether, a score is generated which helps provide recommendations regarding further work up for pulmonary embolism, whether that is a D-dimer or advanced imaging with a CT angiogram.^12^ Once a patient is low risk by Well’s, the PERC score can be applied to clinically rule out a PE. Vital signs, including pulse oximetry, play a large role in a provider’s index of suspicion for PE. Pulse oximetry uses differences in infrared (oxyhemoglobin) and red (deoxyhemoglobin) light absorption to indirectly measure blood oxyhemoglobin saturation and **skin pigmentation** can affect the device’s accuracy.^13^ Once diagnosed, PE is treated with either anticoagulation if there are no signs of circulatory collapse or thrombolytics in the case of hemodynamic instability.

Disposition for pulmonary embolism can be assessed using a PESI score (PE severity index) which incorporates patient demographics, historical factors, hemodynamics and exam findings to aid in admission for anticoagulation versus discharge on anticoagulation. Before discharging a patient on a disease-treating medication, one should verify the pricing, insurance considerations, and feasibility of patients being able to fill their prescription to prevent increased morbidity and mortality.

Diversity, Equity and Inclusion (DEI) elements to be discussed are:

1. Racial Bias in Pulse Oximetry: The pulse oximeter uses differences in infrared (oxyhemoglobin) and red (deoxyhemoglobin) light absorption to indirectly measure blood oxyhemoglobin saturation. Poor circulation, **skin pigmentation**, skin thickness, skin temperature, fingernail polish can all affect the device’s accuracy. Pigmentation likely changes the relationship between amount of light transmitted and the oxygen Level.
2. GFR reporting in African-Americans and Non-African-Americans: Race was initially factored into the GFR calculation because studies showed that African- Americans, on average, have higher creatinine Levels, believed to be due to larger muscle mass and dietary differences. However, this assumption does not apply to every individual. In response, the National Kidney Foundation (NKF) and American Society of Nephrology (ASN) created a joint task force to develop a race-free GFR formula. Clinically, this issue has significant consequences: for instance, a non-Black patient with a creatinine of 2.8 may have a lower GFR (18), making them more likely to qualify for a kidney transplant, while a Black patient with the same creatinine Level may have a higher GFR (21), potentially delaying transplant consideration.
3. Social Determinants of Health: Barriers to filling prescriptions such as cost, transportation, distance to pharmacy, lack of insurance, access to primary care for continuation of medication after initial prescription and health literacy are justifications for hospitalizing of patients.

## Figures and Tables

**Table:** 

**Vitals:**	HR 109	BP 188/99	RR 24	Temp 98.9°F	O_2_Sat 95%

**Table:** 

	Milestone	Did not achieve level 1	Level 1	Level 2	Level 3
**1**	**Emergency Stabilization (PC1)**	□ **Did not achieve Level 1**	□ **Recognizes abnormal vital signs**	□ **Recognizes an unstable patient, requiring intervention** **Performs primary assessment** **Discerns data to formulate a diagnostic impression/plan**	□ **Manages and prioritizes critical actions in a critically ill patient** **Reassesses after implementing a stabilizing intervention**
**2**	**Performance of focused history and physical (PC2)**	□ **Did not achieve Level 1**	□ **Performs a reliable, comprehensive history and physical exam**	□ **Performs and communicates a focused history and physical exam based on chief complaint and urgent issues**	□ **Prioritizes essential components of history and physical exam given dynamic circumstances**
**3**	**Diagnostic studies (PC3)**	□ **Did not achieve Level 1**	□ **Determines the necessity of diagnostic studies**	□ **Orders appropriate diagnostic studies** **Performs appropriate bedside diagnostic studies/procedures**	□ **Prioritizes essential testing** **Interprets results of diagnostic studies** **Considers risks, benefits, contraindications, and alternatives to a diagnostic study or procedure**
**4**	**Diagnosis (PC4)**	□ **Did not achieve Level 1**	□ **Considers a list of potential diagnoses**	□ **Considers an appropriate list of potential diagnosis** **May or may not make correct diagnosis**	□ **Makes the appropriate diagnosis** **Considers other potential diagnoses, avoiding premature closure**
**5**	**Pharmacotherapy (PC5)**	□ **Did not achieve Level 1**	□ **Asks patient for drug allergies**	□ **Selects an appropriate medication for therapeutic intervention, considering potential adverse effects**	□ **Selects the most appropriate medication(s) and understands mechanism of action, effect, and potential side effects** **Considers and recognizes drug-drug interactions**
**6**	**Observation and reassessment (PC6)**	□ **Did not achieve Level 1**	□ **Reevaluates patient at least one time during the case**	□ **Reevaluates patient after most therapeutic interventions**	□ **Consistently evaluates the effectiveness of therapies at appropriate intervals**
**7**	**Disposition (PC7)**	□ **Did not achieve Level 1**	□ **Appropriately selects whether to admit or discharge the patient**	□ **Appropriately selects whether to admit or discharge** **Involves the expertise of some of the appropriate specialists**	□ **Educates the patient appropriately about their disposition** **Assigns patient to an appropriate level of care (ICU/Tele/Floor)** **Involves expertise of all appropriate specialists**
**22**	**Patient centered communication (ICS1)**	□ **Did not achieve level 1**	□ **Establishes rapport and demonstrates empathy to patient (and family)** **Listens effectively**	□ **Elicits patient’s reason for seeking health care**	□ **Manages patient expectations in a manner that minimizes potential for stress, conflict, and misunderstanding.**
**23**	**Team management (ICS2)**	□ **Did not achieve level 1**	□ **Recognizes other members of the patient care team during case (nurse, techs)**	□ **Communicates pertinent information to other healthcare colleagues**	□ **Communicates a clear, succinct, and appropriate handoff with specialists and other colleagues** **Communicates effectively with ancillary staff**

**Table:** 

**#1**	**Patient Information Form**
**#2**	**Complete blood count (CBC)**
**#3**	**Basic metabolic panel (BMP)**
**#4**	**Urinalysis**
**#5**	**Troponin**
**#6**	**D-dimer**
**#7**	**NT-proBNP**
**#8**	**Chest X-ray**
**#9**	**CT-angiogram chest (PE Protocol)**
**#10**	**ECG**
**#11**	**Return of patient to Emergency Department with massive pulmonary embolism**

**Table:** 

**Patient’s Name:**	**John Smith**
**Age:**	**65**
**Gender:**	**Male**
**Chief Complaint:**	**Chest pain**
**Person Providing History:**	**Patient**
**Vital Signs:**	
**Temp:**	**98.9° F**
**BP:**	**188/99**
**P:**	**109**
**RR:**	**24**
**Pulse Ox:**	**95**
**Weight:**	**180 lbs**

**Table:** 

**WBC**	**9.5 × 10** **^3^** **/μL**
**Hgb**	**13.9 g/dL**
**Hct**	**50%**
**Platelets**	**260 ×10** **^3^** **/μL**
**Differential**	
**Neutrophils**	**64%**
**Lymphocytes**	**19%**
**Monocytes**	**13%**
**Eosinophils**	**2%**
**Bands**	**2%**

**Table:** 

**Na**	**135 mEq/L**
**K**	**4.0 mEq/L**
**Cl**	**99 mEq/L**
**CO2**	**24 mEq/L**
**BUN**	**25 mg/dL**
**Cr**	**1.3 mg/dL**
**Glucose**	**150 mg/dL**
**GFR (Black):**	**49 mL/min/1.73m** **^2^**
**GFR:**	**45 mL/min/1.73m** **^2^**

**Table:** 

**Appearance**	**Normal**
**Color**	**Clear**
**Glucose**	**0**
**Ketones**	**none**
**Sp Gravity**	**1.009**
**Blood**	**none**
**pH**	**6.2**
**Protein**	**none**
**Nitrite**	**negative**
**Leukocyte**	**negative**
**WBC**	**0**
**RBC**	**0**
**Squamous Cells**	**1**
**Bacteria**	**0**

**Table:** 

**Troponin T:**	**< 0.01 ng/mL**

**Table:** 

**D-Dimer:**	**2.1 μ/mL (< 0.5 μ/mL)**

**Table:** 

**NT proBNP:**	**93 pg/mL (<125 pg/mL)**

**Figure:**
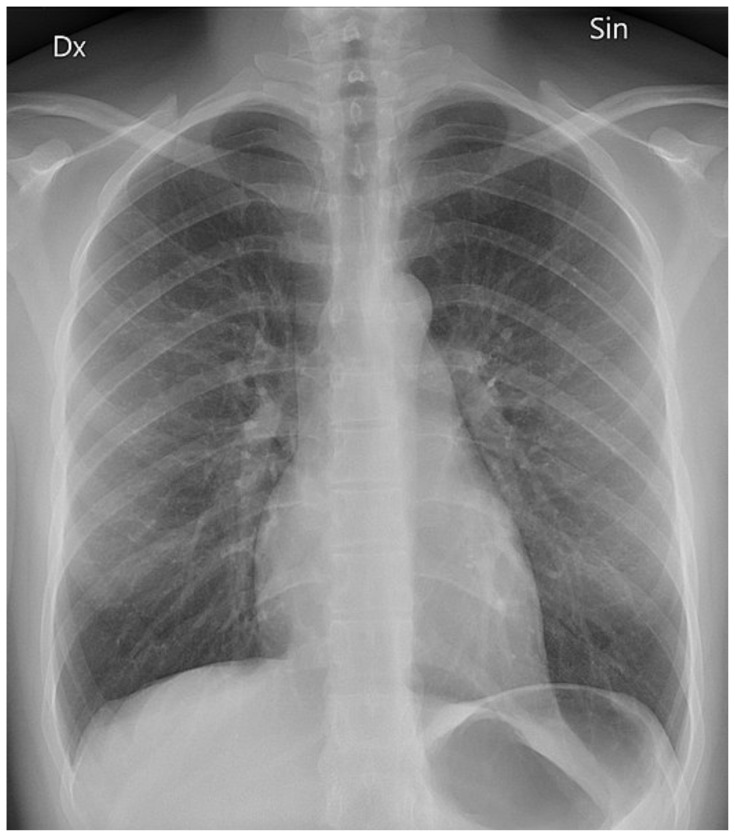


**Figure:**
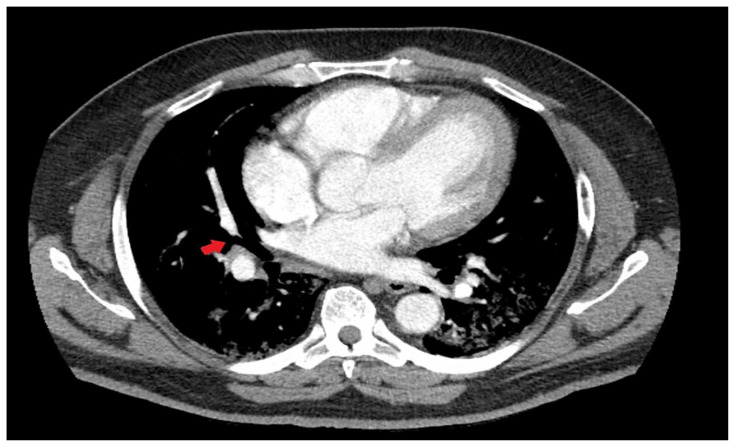


**Figure:**
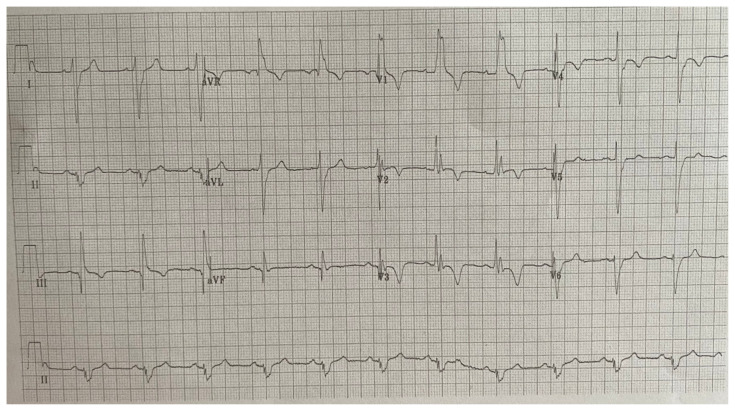


## FOR EXAMINER ONLY

### Oral Case Summary

**Diagnosis: Pulmonary Embolism**

**Case Summary:** A 65-year-old African-American male truck driver develops pleuritic chest pain, associated upper back pain, and shortness of breath from a pulmonary embolism. He has acute coronary syndrome risk factors. He is hypoxic, tachycardic and tachypneic, but otherwise, a normal physical exam. A chest pain workup is obtained. Students will discuss PERC & Well’s Score to decide on D-Dimer vs. computed tomography angiography of chest (CTA). Students will interpret an electrocardiogram (ECG), chest x-ray, and CTA. Students will discuss management and disposition of the patient. He will be discharged on rivaroxaban. He will return to the emergency department 10 days later, very acutely ill from a massive pulmonary embolism. He states he was unable to afford the prescription. This is provided to the learner with stimulus 11. The case then ends and transitions into debriefing.

**Order of Case:** Learners should quickly identify the patient’s abnormal vital signs and obtain IV access while placing the patient on cardiac monitoring and continuous pulse oximetry. If they fail to do so early in the case, the nurse will cue the patient. Learners should also quickly ask for an ECG, and if failing to do so, will also be prompted by the nurse. After obtaining a history and examining the patient, learners should ask for labs and imaging, which correlates with stimuli 2–10, and discuss the patient's Well’s Score before obtaining a CT angiogram chest. If the learners obtain a D-dimer, it will be elevated. Pulmonary embolism is then diagnosed. At this point, learners may propose various anticoagulation and disposition strategies. This is a valuable discussion that can be facilitated by the instructor. Ultimately, stimulus 11 will then be provided to the learner which indicates the patient is discharged with oral anticoagulation, and later returns to the ED with a massive pulmonary embolism. The case ends with prompting the learner to discuss factors that may have led to the patient’s bounce-back to the emergency department and transitions to debriefing on occult hypoxemia and addressing social determinants of health during discharge planning. Of note, stimulus 11 is meant to be a springboard for discussion of barriers to care that could have been addressed on the initial ED visit, and not the management of massive pulmonary embolism. However, if time permits, this can certainly be discussed with the learners at the discretion of the facilitator.

**Disposition:** Discharged home with anticoagulation prescription.

**Critical Actions:**

1. Place patient on monitor, obtain IV access, obtain vitals
2. Obtain an ECG
3. Demonstrate knowledge of PERC and Well’s Score
4. Order and interpret appropriate initial diagnostic tests (lab, imaging)
5. Management of pulmonary embolism
6. Review and discuss social determinants of health and DEI elements
  - Racial bias in pulse oximetry
  - Glomerular filtration rate (GFR)
  - Inability to fill prescription and social determinants of health
  - Presenting this Patient: “65-year-old African-American male…”

### Historical Information

**Chief Complaint:** Chest pain

**History of present illness:** A 65-year-old male presents with chest pain that started yesterday morning. Pain is located in the middle of the chest, left side and radiates to the left upper back. The chest pain is pleuritic, sharp and stabbing. This is the first episode. The patient was eating breakfast when it occurred. Pain was sudden in onset and has been constant. This pain is not exertional, positional or reproducible. There is associated shortness of breath on exertion and at rest. There is no lower extremity edema. No associated palpitations, nausea, vomiting, diaphoresis, fever, cough, hemoptysis, sore throat, rhinorrhea, or myalgias. There were no recent upper respiratory infection symptoms. The patient denies calf pain or swelling, venous thromboembolism (VTE) history or risk factors (immobilizations, recent surgeries, cancer, oral contraceptive (OCP) history, hypercoagulable disorders).

**Past Medical History:** Hypertension, hyperlipidemia, Type 2 diabetes

**Past Surgical History:** None

**Patient’s Medications:** Lisinopril, simvastatin, metformin.

**Allergies:** None

**Social history:**

- Smoking: 1 pack per day for 45 years
- Drug use: None
- Employment: Truck driver. Just completed a long route last week.

**Family history:** Patient not sure.

### Physical Exam Information

**Weight:** 180 lb

**General appearance:** Uncomfortable appearing but no acute distress. No diaphoresis.

**Primary survey:**

- **Airway:** patent, speaks in full sentences
- **Breathing:** tachypnea but bilateral breath sounds present
- **Circulation:** strong distal pulses

**Physical examination:**

- **General appearance:** Uncomfortable appearing but no acute distress. No diaphoresis.
- **HEENT:**
  ○ **Head:** Normocephalic, atraumatic
  ○ **Eyes:** Pupils equal and reacting to light (PEARL), extraocular movements intact (EOMI)
  ○ **Ears:** Normal
  ○ **Nose:** Normal
  ○ **Oropharynx/Throat:** Normal
- **Neck:** No Jugular venous distention (JVD). No carotid bruit. No thyromegaly.
- **Chest:** Tachypnea. Hypoxia. No respiratory distress. No increased work of breathing. No stridor. Lung fields clear throughout on auscultation.
- **Cardiovascular:** Normal S1, S2. No rubs, murmurs or gallops. Tachycardic. Regular rhythm. 2+ radial pulses, bilaterally.
- **Abdominal/GI:** Soft, non-distended, non-tender. No rebound or guarding. Not peritoneal.
- **Genitourinary:** Deferred.
- **Rectal:** Deferred.
- **Extremities:** No calf pain or calf swelling. No palpable cord. Negative Homan’s sign. No bony tenderness. Range of movement (ROM) is full and painless.
- **Back:** No tenderness to palpation. No cervical tenderness. No rashes.
- **Neuro:** AAOx3. Lucid, follows commands. No slurred speech. Strength 5/5 in upper and lower extremities. Sensation to light touch is intact throughout. No pronator drift. No dysmetria on finger to nose. Gait steady and without difficulty.
- **Skin:** No rashes.
- **Lymph:** No lymphadenopathy.
- **Psych:** Acting appropriately. No evidence of intoxication.

### Critical Actions and Cueing Guidelines

1. **Critical Action 1:** *Place patient on MONITOR, Obtain IV ACCESS, Obtain VITALS*
  - **Cueing Guidelines:** Nurse asks, “Doctor, would you like me to get anything started?”
2. **Critical Action 2:** *Obtains an EKG*
  - **Cueing Guidelines:** Nurse asks, “Doctor, is there a test you would like me to immediately obtain?”
3. **Critical Action 3:** *Demonstrate knowledge of PERC & Well’s Score*
  - **Cueing Guidelines: “**Any clinical decision rules we can use to decide on testing?”
4. **Critical Action 4:** *Order and interpret appropriate initial diagnostic tests (lab, imaging)*
  - **Cueing Guidelines:** Patient asks, “Could it be my heart or a blood clot?”
5. **Critical Action 5:** *Management of Pulmonary Embolism*
  - **Cueing Guidelines:**
    - Patient asks, “So how do we treat this clot?”
    - Patient states, “I would really like to avoid staying in the hospital.”
6. **Critical Action 6:** *Identify Social Determinants of Health and DEI Elements*
  - **Cueing Guidelines:** “Can anyone identify any possible racial biases in this case?”
    - **Racial Bias in Pulse Oximetry:**
      1. “How does the pulse oximeter work?”
      2. “Does skin pigmentation affect the pulse oximeter result?”
    - **Glomerular Filtration Rate (GFR):**
      1. “Why are there 2 GFRs reported?”
    - **Inability to fill prescription and Social Determinants of Health:**
      1. “Why do you think he did not fill the prescription?”
      2. “Does the patient have access to a primary care provider?”
      3. “What resources are you aware of to help navigate barriers of care?”
    - **Presenting this Patient:** “65-year-old African-American male…”
      1. “What is the historical context of mentioning race in the presentation of HPI?”

## ORAL BOARDS ASSESSMENT

*The Silent Saboteur*

Learner: _________________________________________

**Critical Actions:**

□ Place patient on monitor, obtain IV access, obtain vitals
□ Obtain an ECG
□ Demonstrate knowledge of PERC and Well’s Score
□ Order and interpret appropriate initial diagnostic tests (lab, imaging)
□ Management of pulmonary embolism
□ Review and discuss social determinants of health and DEI elements
  □ Racial bias in pulse oximetry
  □ Glomerular filtration rate (GFR)
  □ Inability to fill prescription and social determinants of health
  □ Presenting this Patient: “65-year-old African-American male…”

**Summative and formative comments:**

**Milestone assessment:**

### Stimulus Inventory

#### Stimulus # 11

Case progression: After reviewing the patient’s labs and imaging, the patient is discharged from the emergency department with a pulmonary embolism treated with oral anticoagulation, only to return one week later in hypoxic respiratory failure and shock. The patient is diagnosed with a massive pulmonary embolism after being unable to fill his prescription for a factor Xa inhibitor.

What barriers to care could this patient have experienced?

## DEBRIEFING AND EVALUATION PEARLS

Common symptoms of pulmonary embolism (PE) include sudden chest pain, shortness of breath, palpitations, and hemoptysis. The Well's score is a clinical decision rule used to assess the probability of a patient having a PE^11^. It aids in identifying patients who require additional investigations, such as imaging with computed tomography pulmonary angiography (CTPA) or V/Q scanning, to confirm or exclude the presence of a PE. A Well’s score is comprised of clinical signs and symptoms of DVT, PE is the most likely diagnosis, tachycardia, recent surgery or immobilization, previous PE or DVT, hemoptysis, and malignancy. Altogether, a score is generated which helps provide recommendations regarding further work up for pulmonary embolism, whether that is a D-dimer or advanced imaging with a CT angiogram.^12^ Once a patient is low risk by Well’s, the PERC score can be applied to clinically rule out a PE. Vital signs, including pulse oximetry, play a large role in a provider’s index of suspicion for PE. Pulse oximetry uses differences in infrared (oxyhemoglobin) and red (deoxyhemoglobin) light absorption to indirectly measure blood oxyhemoglobin saturation and **skin pigmentation** can affect the device’s accuracy.^13^ Once diagnosed, PE is treated with either anticoagulation if there are no signs of circulatory collapse or thrombolytics in the case of hemodynamic instability.

Disposition for pulmonary embolism can be assessed using a PESI score (PE severity index) which incorporates patient demographics, historical factors, hemodynamics and exam findings to aid in admission for anticoagulation versus discharge on anticoagulation. Before discharging a patient on a disease-treating medication, one should verify the pricing, insurance considerations, and feasibility of patients being able to fill their prescription to prevent increased morbidity and mortality.

Diversity, Equity and Inclusion (DEI) elements to be discussed are:

1. Racial Bias in Pulse Oximetry: The pulse oximeter uses differences in infrared (oxyhemoglobin) and red (deoxyhemoglobin) light absorption to indirectly measure blood oxyhemoglobin saturation. Poor circulation, **skin pigmentation**, skin thickness, skin temperature, fingernail polish can all affect the device’s accuracy. Pigmentation likely changes the relationship between amount of light transmitted and the oxygen Level.
2. GFR reporting in African-Americans and Non-African-Americans: Race was initially factored into the GFR calculation because studies showed that African-Americans, on average, have higher creatinine Levels, believed to be due to larger muscle mass and dietary differences. However, this assumption does not apply to every individual. In response, the National Kidney Foundation (NKF) and American Society of Nephrology (ASN) created a joint task force to develop a race-free GFR formula. Clinically, this issue has significant consequences: for instance, a non-Black patient with a creatinine of 2.8 may have a lower GFR (18), making them more likely to qualify for a kidney transplant, while a Black patient with the same creatinine Level may have a higher GFR (21), potentially delaying transplant consideration.
3. Social Determinants of Health: Barriers to filling prescriptions such as cost, transportation, distance to pharmacy, lack of insurance, access to primary care for continuation of medication after initial prescription and health literacy are justifications for hospitalizing of patients.

## References

[1] Centers for Disease Control and Prevention Social Determinants of Health at CDC.

[2] Skochelak SE, Stack SJ (2017). Creating the medical schools of the future. Acad Med.

[3] Pollock DA, Lowery DW, O’Brien PM (2001). Emergency medicine and public health: new steps in old directions. Ann Emerg Med.

[4] Balch B Medical students use momentum of anti-racism movement to advocate for change. AAMC News.

[5] Association of American Medical Colleges Diversity, equity, and inclusion competencies across the learning continuum. AAMC, Org.

[6] Bernstein SL, Haukoos JS (2008). Public health, prevention, and emergency medicine: a critical juxtaposition. Acad Emerg Med.

[7] Moffett SE, Shahidi H, Sule H, Lamba S (2019). Social determinants of health curriculum integrated into a core emergency medicine clerkship. MedEdPORTAL.

[8] Von Chong A, Terosiet M, Histace A, Romain O (2019). Towards a novel single-LED pulse oximeter based on a multispectral sensor for IoT applications. Microelectronics Journal.

[9] Sjoding MW, Dickson RP, Iwashyna TJ, Gay SE, Valley TS (2020). Racial bias in pulse oximetry measurement. N Engl J Med.

[10] (2020). Virtual Oral Exam Sample Standard Case [video online].

[11] Gibson NS, Sohne M, Kruipe M (2008). Further validation and simplification of the Wells clinical decision rule in pulmonary embolism. Thromb haemost.

[12] Kline J, Tintinalli JE, Ma OJ, Yealy DM (2020). Venous thromboembolism. Tintinalli’s Emergency Medicine: A Comprehensive Study Guide.

[13] Valbuena VS, Barbaro RP, Claar D (2022). Racial bias in pulse oximetry measurement among patients about to undergo extracorporeal membrane oxygenation in 2019–2020: A retrospective cohort study. Chest.

[14] Haggstrom M Normal posterior/anterior (PA) chest radiograph (X-ray). Wikimedia Commons.

[15] Vedamurthy D, Wang E, Wang H How to manage submassive pulmonary embolism. The Hospitalist.

[16] Burns E, Buttner R ECG changes in pulmonary embolism. Life in the Fastlane website.

